# Enhanced Optical and Electrical Properties of IGZO/Ag/IGZO for Solar Cell Application via Post-Rapid Thermal Annealing

**DOI:** 10.3390/nano14221841

**Published:** 2024-11-18

**Authors:** Chanmin Hwang, Taegi Kim, Yuseong Jang, Doowon Lee, Hee-Dong Kim

**Affiliations:** 1 Department of Semiconductor Systems Engineering, Convergence Engineering for Intelligent Drone, Institute of Semiconductor and System IC, Sejong University, 209, Neungdong-ro, Gwangjin-gu, Seoul 05006, Republic of Korea; hcm4808@sju.ac.kr (C.H.); ktg2356@sju.ac.kr (T.K.); nr7782942@sju.ac.kr (Y.J.); 2 Division of Electrical, Electronic and Control Engineering, Kongju National University, Cheonan 31080, Republic of Korea; doowon.lee@kongju.ac.kr

**Keywords:** transmittance, TCO, RTA, solar cell application

## Abstract

In this paper, we optimized IGZO/Ag/IGZO (IAI) multilayer films by post-rapid thermal annealing (RTA) to enhance the electrical conductivity and optical transmittance in visible wavelengths for solar cell applications. Our optimized device showed an average transmittance of 85% in the visible range, with a lowest sheet resistance of 6.03 Ω/□ when annealed at 500 °C for 60 s. Based on these results, we assessed our device with photo-generated short circuit current density (J_SC_) using a solar cell simulator to confirm its applicability in the solar cell. IAI multilayer RTA at 500 °C for 60 s showed a highest J_SC_ of 40.73 mA/cm^2^. These results show that our proposed IAI multilayer film, which showed a high optical transparency and electrical conductivity optimized with post RTA, seems to be excellent transparent electrode for solar cell applications.

## 1. Introduction

Transparent conductive oxide (TCO), which is widely used in optoelectronic device industries, is a crucial technology for thin-film solar cell applications, with its high optical transparency in the visible range and electrical conductivity [1]. In solar cell applications, since TCO acts as an electrode transferring the charge carrier, the TCO layer should have a high conductivity to avoid parasitic absorption and electrical losses [2]. In order to meet these electrical requirements, electrodes in solar cell applications should have a sheet resistance of at least 80 Ω/□ [3]. Also, TCO plays a role as an optical window that allows light to enter the absorber layer of the solar cell with transmittance and low reflection and absorption properties. In particular, the improvement of TCO transmittance in the visible range is necessary for solar cell efficiency. Solar irradiance shows a peak intensity of around 500 nm and, for this reason, the maximum conversion efficiency is found in the visible wavelength range [4]. This indicates that targeting the maximum efficiency in the visible range is essential in the design of solar cell applications [5]. Therefore, research on transparent electrodes that improve both high conductivity and transmittance in the visible range is necessary from the point of view of solar cell efficiency.

As mentioned, TCO has a high transmittance, but its sheet resistance is relatively high for solar cell applications. To address its high sheet resistance, a TCO/metal/TCO (OMO) structure, which has a thin metal film between the TCO layer, can be an alternative. Compared with single-layer TCO, the OMO triple layer can reduce the reflection from the metal film in the visible range and enhance electrical conductivity. To achieve metallic conductivity while minimizing the resulting loss of transmittance, various TCO and metal films with thickness dependence have been reported [6]. Indium-tin oxide (ITO) is a commonly used TCO material in OMO structures due to its transparency in the visible range and high conductivity compared with other TCOs. However, ITO has several problems such as toxicity and relatively high prices due to the limited supply of indium [7]. Because of these drawbacks of ITO, several zinc oxide (ZnO)-based alternatives have been researched, such as ZnO, aluminum zinc oxide (AZO), and indium gallium zinc oxide (IGZO) [8]. ZnO has a high optical transparency in the visible range, wide band gap, and inexpensive costs. However, due to the low carrier concentration of non-doped ZnO, it has a high resistivity [9]. Although AZO has a higher conductivity compared with ZnO, AZO shows a mismatch in its thermal expansion coefficient compared with a glass substrate [10]. Since IGZO can compensate for the other TCO disadvantages we mentioned, IGZO is a suitable material for solar cell applications with OMO structures due to its relatively low price, high carrier mobility, non-toxicity, optical transparency, and thermal stress stability [1,10,11]. Table 1 summarizes the research on the material properties of TCOs.

In this study, it was important to increase conductivity utilizing metal film, but since the purpose was to apply it to solar cell applications, it was also necessary to consider the transmittance of the metal film. Various metals have been researched for OMO structures, such as Ag, Al, Pt, and Cu [7]. Among them, Ag is considered to be the best metal film candidate for OMO structures in solar cell applications due to its high conductivity and transparency in the visible range compared with other metals. For these reasons, we decided that IGZO/Ag/IGZO (IAI) is an ideal OMO structure for solar cell applications due to its electrical and optical properties. Table 2 shows the optoelectrical properties of IGZO-based OMO structures. Additionally, there is a trade-off depending on the thickness between transmittance and conductivity, and the thickness dependance of IAI has been extensively studied. Consequently, it is considered that a thickness of about 41/14/41 nm IAI has the best electrical and optical properties [15].

To further enhance the electrical and optical properties of IAI for solar cell applications, we utilized the rapid thermal annealing (RTA) method. Li et al. reported that using RTA on TCO materials causes an increase in grain size, resulting in lowering interfacial scattering. Also, the main issue of the low conductivity in TCO can be solved with RTA, leading to an improved hole mobility and carrier concentration [18]. In this paper, we obtain the enhanced optical and electrical properties of IAI films under varying RTA conditions. In addition, we discuss the electrical and optical properties of IAI films as the annealing temperature varied from RT to 500 °C for 60 s. Finally, we optimized IAI multilayers with post RTA to enhance the conductivity and transmittance and assessed the their applicability in solar cell applications through simulation.

## 2. Materials and Methods

The quartz substrates were cleaned with acetone, methanol, and deionized water for 10 min, respectively. The IGZO was deposited on quartz substrates for 41 nm using radio frequency (RF) sputtering (Korea Vacuum tech, KVS-2000L, Gimpo-si, Gyeonggi-do, Republic of Korea) at 100 W with 20 sccm of Ar. The base pressure and working pressure of the sputtering chamber during IGZO deposition were under 5 and 20 mTorr. After that, the 14 nm thick Ag was deposited using electron beam evaporation (Shvac, SHE-6D-350T, Seoul, Republic of Korea). Subsequently, the top IGZO layer was deposited using the same conditions as those for the bottom IGZO layer. With the same deposition method, a single layer of IGZO 200 nm was deposited on a quartz substrate for comparison. After the deposition of the IGZO/Ag/IGZO (IAI) multilayer, RTA was performed using MILA-4000 (ULVAC, Chigasaki, Japan) in N_2_ atmosphere gas, varying the temperature from 300 to 500 °C for 60 s. In order to analyze the electrical properties of the IGZO/Ag/IGZO multilayers, a 4-point probe (AIT, CMT-SR2000 N, Suwon-si, Gyeonggi-do, Republic of Korea) was used. A current of 10 mA was applied to the four probes, and the voltage was measured to obtain the resistance. A correlation factor (4.55) was applied to the sample size to calculate it in sheet resistance. The measurements were conducted 10 times in each condition to obtain an average of the sheet resistance. The impedance of IAI was measured using Keithley 4200 SCS (Keithley Instruments, Solon, OH, USA). In addition, we measured the IAI surface roughness using Atomic Force Microscopy (AFM, Park System Corp., Suwon-si, Gyeonggi-do, Republic of Korea). To observe the optical properties, transmittance and reflectance were measured by a UV-Vis spectrophotometer (Varian, FC-PH10, Santa Clara, CA, USA) in the spectral range from 200 to 1100 nm. In order to evaluate the potential of the IAI multilayers via post RTA for solar cell applications, a PV light house simulation was conducted. The simulation structure was Air/IAI/Si. The light intensity was set to 44 mA/cm^2^. The refractive index was analyzed with the measured data by an Ellipsometer (J. A Woollam Co., Ltd., Alpha SE, Lincoln, NE, USA).

## 3. Results and Discussion

**Figure 1:**
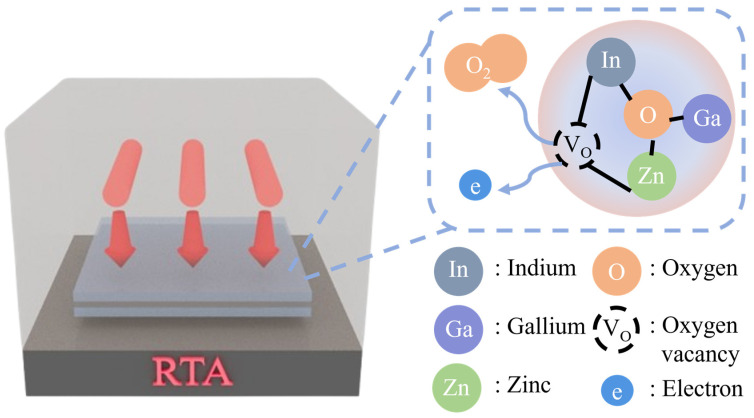
The RTA process for the IGZO/Ag/IGZO multilayer, and the right diagram describes the generated oxygen vacancies from the IGZO inside by RTA.

**Figure 2:**
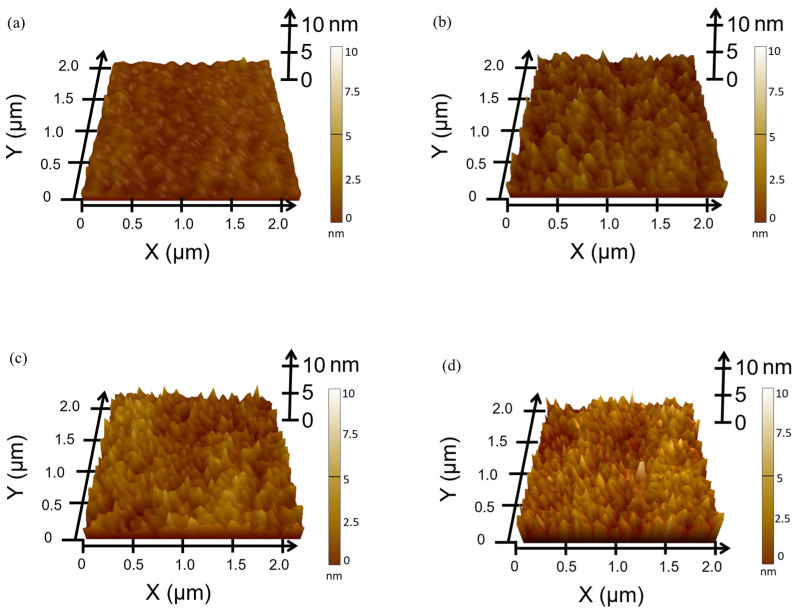
AFM topology images of IGZO/Ag/IGZO after RTA, (**a**) RT, (**b**) 300, (**c**) 400, and (**d**) 500 °C for 60 s.

Figure 1 shows the RTA process for the IAI multilayers. In the RTA process, IGZO and Ag molecules are annealed from outside. Using RTA on an IAI multilayer generates an oxygen vacancy in IGZO and enhances the crystallinity of each layer [10,19]. Thus, the electrical and optical properties can be enhanced with the RTA process. First, since the surface roughness is an important factor affecting the electrical and optical properties, we analyzed the effect of RTA on the surface morphologies of the IAI multilayer films. Thus, AFM measurements were conducted under varying annealing conditions, and we obtained topographical images and the root mean square roughness (R_q_), as shown in Figure 2 and Table 3. The R_q_ value was measured within the designated 2 × 2 μm^2^ area. As the annealing temperature of RTA increased from RT to 500 °C, the R_q_ of the IAI multilayer was increased. In detail, the initial R_q_ value was 0.525 nm, which increased with the RTA temperature, reaching the highest R_q_ value of 0.936 nm in the 500 °C annealing condition. This increase in the surface roughness of the IAI multilayer was due to the crystallization of the IGZO layer, resulting in an increased grain size [20]. The initial phase of the IGZO layer was amorphous, showing low roughness and R_q_ values. After the RTA process, as seen from the AFM topographies, the structure grew with temperature. This was caused by the crystallization of the IGZO layer and was associated with having higher R_q_ values. Also, the IGZO layer could effectively suppress the surface agglomeration of the Ag layer, therefore maintaining morphological stability at high temperatures. A single Ag film faces a limitation in the annealing process, since it can agglomerate easily at a high temperature, resulting in degradation in the electrical and optical properties. The agglomeration of the Ag film is dominated by the surface diffusion of Ag atoms, in which the suppression of surface diffusion can be an effective method to prevent agglomeration [21]. In this study, we observed that IGZO can suppress the agglomeration of the Ag layer effectively. Thus, IAI multilayers can be annealed effectively to crystallize the IGZO layer without the agglomeration of the Ag layer. To summarize our material analysis, we observed that an increased annealing temperature caused the crystallization of IGZO layer, resulting in an increase in surface roughness [22].

**Figure 3:**
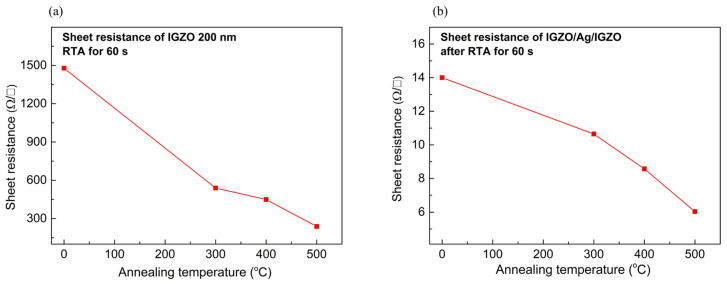
The sheet resistance of (**a**) IGZO and (**b**) IGZO/Ag/IGZO multilayers after varying temperature conditions.

Next, to evaluate the electrical properties of the IAI multilayer as a function of the annealing temperature, we measured the sheet resistance. After the RTA process, the sheet resistance of the IGZO and IAI multilayers decreased as the annealing temperature increased. In Figure 3a, the IGZO layer showed a minimum sheet resistance value of 238 Ω/□ at 500 °C. This tendency to decrease the sheet resistance of the IGZO layer with an increasing annealing temperature can be explained by the improved crystallization of the IGZO layer and increases in free electrons caused by oxygen vacancies [23]. According to Equation (1), two electrons are generated per one oxygen vacancy since the oxygen atoms in the IGZO layer can leave their original sites. Also, these charge transition levels for oxygen vacancies are slightly below the minimum conduction band, which indicates the formation of a shallow donor level [23]. Therefore, it is expected that the formation of oxygen vacancy can be enhanced by a higher annealing temperature, resulting in an increase in the carrier concentration and a decrease in the sheet resistance.

(1) $$O_{o}^{x}=\frac{1}{2}O_{2(g)}+V_{o}^{\cdot \cdot }+2e^{-}$$

**Figure 4:**
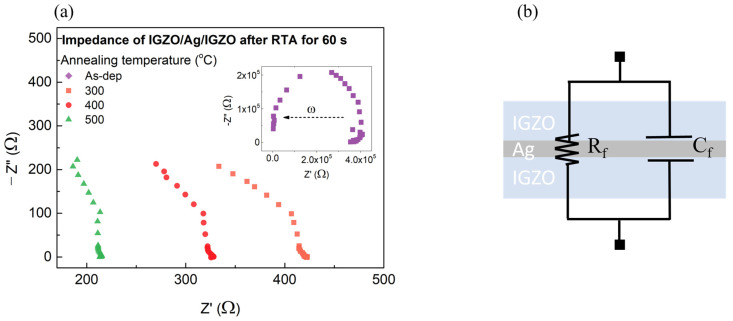
(**a**) Impedance of IGZO/Ag/IGZO after RTA RT to 500 °C for 60 s and (**b**) equivalent circuit of IGZO/Ag/IGZO.

However, although the RTA process can decrease the sheet resistance of the IGZO layer, a sheet resistance of 238 Ω/□ is too high to apply in solar cell applications [3]. In order to address the high resistance of IGZO, as mentioned, we suggested an IAI structure. Figure 3b shows the effect of post-RTA on the sheet resistance of the IAI multilayer. Due to the high conductivity of the Ag layer, the initial IAI film showed a low sheet resistance of 14 Ω/□. The sheet resistance decreased with an increasing annealing temperature, which can be attributed to the increased oxygen vacancies and improved crystallization of the IGZO layer. Notably, it showed a lowest sheet resistance of 6.03 Ω/□ annealed at 500 °C for 60 s. To further investigate the electrical properties of the IAI multilayer, we measured the impedance of IAI in the frequency range from 1 kHz to 10 MHz. Figure 4a shows the impedance of the IAI multilayer with annealing temperature. Figure 4b shows an equivalent circuit diagram of the IAI multilayer, consisting of film resistance R_f_ and film capacitance C_f_. Each IGZO layer has a parallel resistance (R_f_)–capacitance (C_f_), while the Ag layer shows metallic resistance, and it is, therefore, assumed to function as a simple conductor. As a result, the IAI multilayer is composed of parallel resistance and capacitance, as shown in Equation (2). We observed that the trend of impedance curves is well matched with the RC parallel circuit.

(2) $$Z\left(\omega \right)=\frac{R_{f}}{1+\omega^{2}C_{f}^{2}R_{f}^{2}}-j\frac{\omega C_{f}R_{f}^{2}}{1+\omega^{2}C_{f}^{2}R_{f}^{2}}$$

The impedance of the real part decreased with an increasing annealing temperature from RT to 500 °C. In the case of the as-deposited IAI multilayer as shown in the inset, the IGZO layer was in an amorphous state, resulting in a high resistivity and large C_f_. Also, as mentioned, the initial IGZO layer showed a high sheet resistance. On the other hand, the IAI multilayer with the post RTA process had a decreased C_f_ value due to the improved conductivity with the crystallization and generation of oxygen vacancies in the IGZO layer. Also, this caused low impedance and minimal variations at higher frequencies as a function of annealing temperature. Since the as-deposited IAI multilayer showed a clear inflection point, we conducted fitting on it, where ω represents the angular velocity. The characteristic frequency at the Debye peak maxima for each RC element is given by Equation (3) [24].

(3) $$f_{max}=\frac{1}{2\pi RC}$$

As a result, we obtained a C_f_ of 438 mF and R_f_ of 3.6 MΩ for the as-deposited IAI multilayer. These results confirm our prior observation that the annealing temperature affected the decrease in C_f_, and the frequency effect became less significant. The impedance of the IAI multilayer can also be attributed to the surface roughness. The roughness of the surface provides a large effective surface area to the electrode, facilitating an increase in charge injection per unit area at the interface [25]. Furthermore, we found a similar trend between the surface roughness and impedance, along with variation with an increasing frequency of the IAI multilayer.

**Figure 5:**
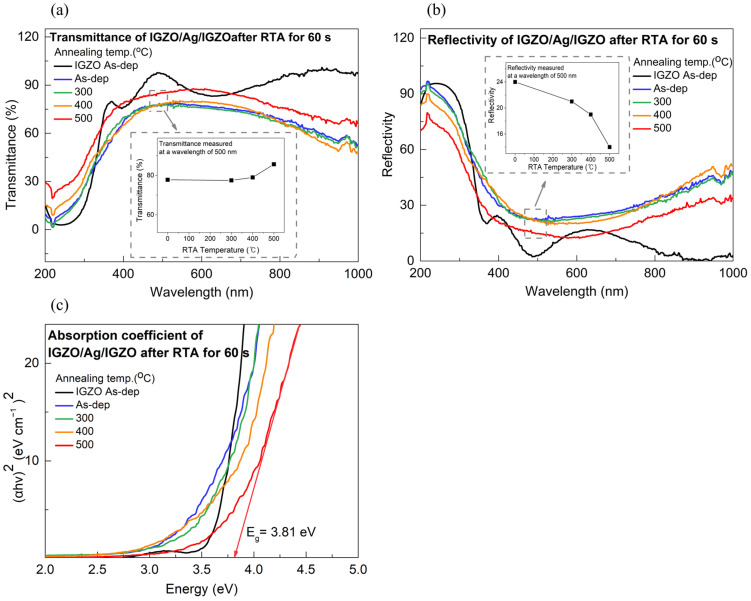
(**a**) Transmittance, (**b**) reflectivity, and (**c**) absorption coefficient of IGZO/Ag/IGZO after RTA RT to 500 °C for 60 s, and each inset depicts the transmittance and reflectivity at 500 nm wavelength with varying annealing temperature.

In order to evaluate the IAI multilayer film as a transparent electrode for solar cell applications, we investigated the optical properties in the wavelength range from 200 to 1100 nm using a UV-Vis spectrophotometer. Figure 5a,b show the transmittance and reflectivity of the IAI multilayer as a function of annealing temperature, respectively, and the insets in each figure show the trends of transmittance and reflectance with annealing temperature at a 500 nm wavelength. The average transmittance in the visible range tended to increase from 76% to 84% with an increasing RTA temperature, which can be attributed to the crystallization of the IGZO layer due to RTA [26]. Initially, the IGZO layer was amorphous, but as the annealing temperature increased, nanocrystalline structures formed within the IGZO layer [27], leading to its crystallization. Therefore, the densification of IGZO particles due to RTA could improve the device’s transmittance by reducing optical scattering, which is caused by grain growth and a decrease in grain boundary density [28]. However, no improvement in transmittance was observed at 300 °C, likely because the relatively low temperature was insufficient for adequate crystallization.

Additionally, when considering the Ag layer, the transmittance trend varied with wavelength depending on the annealing conditions. Unlike the visible range, the transmittance tended to decrease in the longer-wavelength region, which may have been due to the optical properties of the Ag layer. The absorption coefficient of Ag is determined by interband electronic transitions [29], and since the absorption coefficient of Ag was higher in the longer-wavelength region, the transmittance of the IAI multilayer decreased in the long-wavelength range after annealing [30]. However, at 500 °C, the transmittance increased in the long-wavelength region. This can be attributed to the dominant effect of IGZO crystallization, rather than the influence of the Ag layer’s absorption coefficient.

As seen from the previous AFM data, the change in Rq after 500 °C was much larger than that at 300 °C and 400 °C, indicating that the crystallization of IGZO occurred significantly in the 500 °C annealing condition. In other words, the crystallization of IGZO was notably enhanced at 500 °C [31], and the increase in transmittance due to this effect was more dominant than the change in the absorption coefficient of the Ag layer.

On the other hand, in the near-ultraviolet region, the transmittance of the IAI multilayer increased after RTA. This phenomenon may have been due to the fact that the absorption coefficient of Ag was smaller in the near-ultraviolet region compared to the long-wavelength region, in addition to the crystallization of IGZO.

Figure 5b shows the reflectivity of the IAI multilayer as a function of annealing temperature. This shows that the reflectivity decreased with annealing temperature, which was caused by the increase in surface roughness. As the surface roughness of the IAI multilayer increased, light was reflected in different directions. This caused light to travel through multiple paths, increasing its interaction with the layer. This effect increased the light’s path within the film, resulting in less light for the IAI multilayer [32]. Based on these optical data, we calculated the absorption coefficients of IAI multilayer. Figure 5c shows a plot of the absorption coefficient versus photon energy. The following formula describes the relationship between the absorption coefficient and energy band gap *E_g_.*

(4) $$\alpha \left(hv\right)\;\sim \;{\left(hv-E_{g}\right)}^{1/2}$$

hv is the photon energy and α is the absorption coefficient. According to the fitting result, the optical band gap of the as-deposited IAI multilayer was ~3.51 eV. Additionally, an increase in the band gap energy was observed as a function of annealing temperature, which showed an energy band gap of ~3.8 eV at 500 °C, as shown in Table 4. This phenomenon was due to the Burstein–Moss effect [33]. With annealing temperature, the carrier concentrations of the IAI multilayer were increased. When the electrons exceed the density of the state at the bottom conduction band, the Fermi level moves toward the conduction band. This leads to electrons needing more energy to transition from the valence band to the conduction band, resulting in the band gap energy being widened.

**Figure 6:**
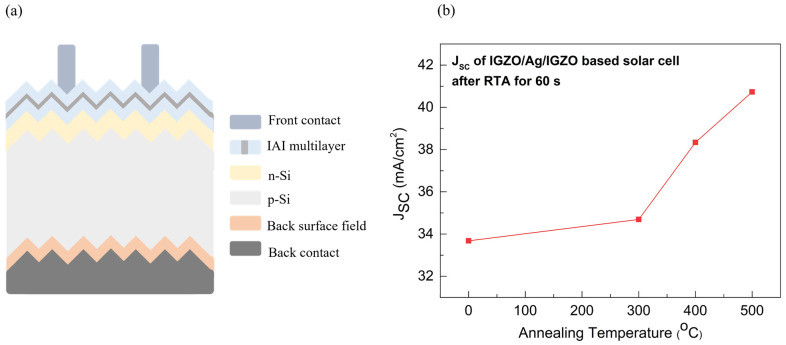
(**a**) Schematic structure of IGZO/Ag/IGZO-based solar cell and (**b**) the photo-generated short circuit current of IGZO/Ag/IGZO-based solar cell as a function of annealing temperature.

Based on these results, in order to evaluate the feasibility of IAI multilayer films as transparent electrodes for solar cell applications, we analyzed a photo-generated short circuit current (J_SC_) absorbed in a Si substrate using a PV light house simulator. Figure 6a shows the schematic structure of an IAI-based solar cell applied in the solar cell simulation. Figure 6b shows the J_SC_ absorbed in the Si substrate as a function of annealing temperature. At the annealing condition of 500 °C or 60 s, it showed a maximum J_SC_ of 40 mA/cm^2^. This trend is similar to the transmittance, reflectivity, and surface roughness previously mentioned. As the transmittance and reflectivity of the IAI multilayer improved, less light was lost to reach the Si substrate, leading to an enhanced optical efficiency. Therefore, our proposed IAI multilayer, optimized with the RTA process, is considered to show a high performance for solar cell applications due to its optical and electrical properties.

## 4. Conclusions

In this study, we proposed an IGZO/Ag/IGZO multilayer film fabricated through a post RTA process to optimize it as a transparent electrode for solar cell applications. After RTA under varying temperatures conditions, we analyzed the enhanced electrical and optical properties of the IAI. The lowest sheet resistance of 6.03 Ω/□ was observed on IAI annealed at RTA at 500 °C for 60 s. Under the same condition, it showed the highest transmittance over 80% in the visible range. Based on these results, we conducted a solar cell simulation, and it showed a high J_SC_ of 40 mA/cm^2^. Therefore, these results indicate that IAI optimized via post RTA has potential for solar cell applications due to its high conductivity and transmittance properties.

## Figures and Tables

**Table 1:** Electrical and optical properties of TCOs, along with their advantages and disadvantages.

Material	Thickness (nm)	R_sq_ (Ω/□)	T (%) (Visible Range)	Advantages	Disadvantages	Ref.
ITO	120	103	~80	Low resistivity, high optical transmittance	High price, toxicity	[7,12]
ZnO	292	373	~80	Inexpensive, non-toxicity	Low carrier concentration	[9,13]
AZO	185	~300	~90	Higher conductivity than ZnO	Mismatch thermal expansion coefficient	[10,14]
IGZO	200	238	87	Inexpensive, higher carrier mobility	Higher resistance than ITO	[10,11]

**Table 2:** Electrical and optical properties of IGZO based OMO multilayer.

Structure	Thickness (nm)	R_sq_ (Ω/□)	T (%) (Visible Range)	Ref.
IGZO/Ag/IGZO	41/14/41	14	76.23	This work
IGZO/Ni/IGZO	50/15/50	47.4	54.8	[16]
IGZO/Cu/IGZO	30/9/30	5.5	28	[17]

**Table 3:** The R_q_ of IGZO/Ag/IGZO, depending on different annealing temperatures.

Annealing Temperature (℃)	R_q_ (nm)
RT	0.525
300	0.646
400	0.715
500	0.936

**Table 4:** The average transmittance in visible range and optical band gap of IAI multilayer, depending on different annealing temperatures.

Annealing Temperature (℃)	Average Transmittance in Visible Range (%)	Optical Band Gap (eV)
RT	76	3.58
300	75.4	3.59
400	77.1	3.69
500	84.9	3.81

## Data Availability

The data are contained within the article.
